# Opportunities and Challenges in Implementation of Multiparameter Single Cell Analysis Platforms for Clinical Translation

**DOI:** 10.1111/cts.12536

**Published:** 2018-03-02

**Authors:** Susan M. Keating, D. Lansing Taylor, Anne L. Plant, E. David Litwack, Peter Kuhn, Emily J. Greenspan, Christopher M. Hartshorn, Caroline C. Sigman, Gary J. Kelloff, David D. Chang, Gregory Friberg, Jerry S. H. Lee, Keisuke Kuida

**Affiliations:** ^1^ CCS Associates San Jose California USA; ^2^ University of Pittsburgh Drug Discovery Institute University of Pittsburgh Pittsburgh Pennsylvania USA; ^3^ Biosystems and Biomaterials Division Materials Measurement Laboratory National Institute of Standards and Technology Gaithersburg Maryland USA; ^4^ Office of In Vitro Diagnostics and Radiological Health Center for Devices and Radiological Health Food and Drug Administration Silver Spring Maryland USA; ^5^ University of Southern California Los Angeles California USA; ^6^ Center for Strategic Scientific Initiatives National Cancer Institute Bethesda Maryland USA; ^7^ National Cancer Institute Bethesda Maryland USA; ^8^ Kite Pharma, Inc. Santa Monica California USA; ^9^ Amgen Thousand Oaks California USA; ^10^ Deciphera Pharmaceuticals LLC Waltham Massachusetts USA

## Abstract

The high‐content interrogation of single cells with platforms optimized for the multiparameter characterization of cells in liquid and solid biopsy samples can enable characterization of heterogeneous populations of cells *ex vivo*. Doing so will advance the diagnosis, prognosis, and treatment of cancer and other diseases. However, it is important to understand the unique issues in resolving heterogeneity and variability at the single cell level before navigating the validation and regulatory requirements in order for these technologies to impact patient care. Since 2013, leading experts representing industry, academia, and government have been brought together as part of the Foundation for the National Institutes of Health (FNIH) Biomarkers Consortium to foster the potential of high‐content data integration for clinical translation.

Precision medicine is moving cancer treatment from etiological and histological parameter‐based treatments to those that target specific key molecular drivers of disease in a time‐resolved fashion. To arrive at actionable end points, representative subsamples of the disease tissue and multiparameter measurements are required to accurately profile tissues at the single cell level to describe the relevant biological unit of disease. Bulk measurements must assume a normal distribution of data points. Thus, given the possible spatial and temporal heterogeneity of tumors with morphological, biochemical, physical, and genetically different subpopulations, coupled with the capacity for cell evolution,^1^, ^2^ the true variation of cellular features in the population may be masked if the measured responses from cells are averaged across cell populations, limiting the ability to detect and target relevant biomarkers.^3^ For example, in solid tumors, the tumor is a “system” within the organ that includes normal cells, cancer cells exhibiting a range of genetic alterations, stromal cells, including fibroblasts, vasculature cells, and immune cells, such as dendritic cells, granulocytes, macrophages, and lymphocytes that are critical to the development, progression, metastasis, and response to therapy, for both standard cytotoxic chemotherapy and immunotherapy. The value in analyzing single cells as biological units of disease to more accurately record the individual and range of cellular responses is increasingly recognized, as is the value of multiparameter single cell analysis that can be obtained using next‐generation technologies. This includes identification of rare genetic mutations within a tumor, better understanding of signaling and metabolic pathways, and prediction of the optimal treatment regimens to prevent tumor regrowth.^4^, ^5^, ^6^ Next‐generation technologies are defined here as methods that permit medium to high throughput, and multiparameter analyses of either fixed or living, single cell morphometric, genomic, proteomic, and/or metabolomics characteristics. Significant challenges include the deconvolution of biological variation from technical uncertainty, the computational analysis of biological variations over time and space, and the integration of single cell genomic, proteomic, and metabolic data.

High‐content platforms were initially built over 2 decades ago for drug discovery screening with a focus on the temporal‐spatial dynamics and heterogeneity among cells by making measurements on each cell in a population.^7^, ^8^ Since they were first introduced, high‐content systems have been further developed to incorporate advances in optical contrast physics, automated microscopy systems, image analysis software, fluorescence‐based reagents, and computational methods. They are often coupled with other platforms, such as next‐generation sequencing for genomic profiling of cells and/or imaging mass cytometry, and are deployed as large‐scale information‐based tools.^9^ The multiparameter information available in these systems is starting to be more fully exploited in *in vitro* and *ex vivo* settings to understand the biology of clinical samples. Furthermore, the definition of high‐content has evolved to include the methods defined above as next‐generation technologies that apply to genomic, proteomic, and/or metabolomic measurements that are based on single cell, usually extensive, multiparameter measurements.

The High‐Content Data Integration (HCDI) working group was formed in early 2013 out of the Foundation for the National Institutes of Health (FNIH) Biomarkers Consortium Cancer Steering Committee (a public‐private partnership) to evaluate emerging technologies (other than those for sequencing) that use advanced acquisition and analysis of single and bulk cell populations for development of tailored signatures that can assist in patient stratification and identification of patient responses to treatment. The HCDI working group has discussed platforms for patient sample single‐cell analysis, such as those listed in **Table** 1, while considering key challenges and critical questions concerning real‐world development and translational potential of these types of platforms, as listed in **Table** 2. In this article, the challenges encountered with newly evolving high‐content measurement systems are illustrated with examples of two platforms, the first, the High Definition Single Cell Analysis (HD‐SCA) workflow, optimized for identifying and characterizing rare cells in liquid biopsy samples and the second, the MultiOmyx Immunofluorescence (MxIF), for enabling “hyperplexed” measurements of proteins and nucleic acids in single cells or in tissues. Creation of effective approaches to the challenges discussed here can enable characterization of heterogeneous populations of cells *ex vivo*, the search for agents to treat cancer and other diseases, and identification of characteristics useful for more precise diagnosis of disease and therapeutic development.

**Table 1:** Examples of new technologies capable of clinical single cell analysis

Technology platform	Developer / sponsor	Description	Data elements
HD‐SCA	Peter Kuhn, University of Southern California / Commercialization: Epic Sciences, San Diego, CA	Imaging platform for multiplex single cell measurements, imaging, immunofluorescence labelling. Individual cells can be picked for DNA sequencing. Slides can also be subjected to laser ablation for imaging mass cytometry for spatial resolution of proteins (Fluidigm, South San Francisco, CA).^52^	Cell images: ○ Cell and nuclear morphology ○ Selected cell and nuclear protein expression (2–3 immunofluorescence label intensity) ○ Multiplex protein expression (4, 5, 6, 7, 8, 9, 10, 11, 12, 13, 14, 15, 16, 17, 18, 19, 20, 21, 22, 23, 24, 25, 26, 27, 28, 29, 30, 31, 32, 33, 34, 35, 36, 37) Single cell DNA sequencing
SMR	Scott Manalis / Massachusetts Institute of Technology	Single‐cell mass accumulation rate real‐time measurements over short time frames (20 min). *Ex vivo* evaluation of patient samples provides evidence as to the range in responses from a patient's tumor cells to a drug, including development of resistance.^53^	Time‐stamped single‐cell mass (changes) Additional, protein expression^54^
MxIF GE commercial name for imaging platform: Cell Dive	GE Global Research and Lans Taylor & Chakra Chennubhotla / University of Pittsburgh	Sequential fluorescent labeling of slides with antibodies, DNA and RNA probes, imaging for “hyperplexed” (>7 biomarkers up to ca. 60) fluorescence imaging for quantitative, single‐cell, and subcellular characterization of analytes in formalin‐fixed paraffin‐embedded tissue coupled with spatial statistical methods to define microdomains.^24^, ^25^	Cell images (immunofluorescence label intensity): ○ Protein expression ○ RNA ○ DNA ○ *In situ* hybridization
SCBC	James Heath / California Institute of Technology	Multiplex quantitative protein expression, secretion, and intracellular signaling, from single cells. Dissociated cells are introduced into microchambers containing miniature antibody‐DNA‐barcoded microarrays. Analyte detection using miniature ELISA measurement and quantitation methods.^42^, ^55^, ^56^	Cell‐based (immunofluorescence label intensity) >20 protein expression
Mass spectrometry imaging	Garry Nolan / Stanford University	Single‐cell analysis utilizing mass spectrometric measurement of metal elements tagged to antibodies. Individual antibody‐bound cell is vaporized, ionized, and analyzed on a mass spectrometer.^57^	Simultaneous quantification of 50 mass tags (markers)
CAFE MiCells	David Andrews / Sunnybrook Research Institute	Automated high‐content image analysis using nontoxic, cell permeable dyes^58^	Visualization of cell states and outcomes of treatment

CAFÉ MiCells, classification and automated feature extraction of micrographs of cells; ELISA, enzyme‐linked immunosorbent assay; HD‐SCA, high definition single cell analysis; MxIF, MultiOmyx; SCBC, single‐cell barcode chip; SMR, suspended microchannel resonator.

**Table 2:** New technology platform translational potential checklist

What are the possible translational or clinical research applications?
Does the method meet an unmet medical need or significantly improve on existing technology? Any competition?
Provide technical description as needed (critical hardware and software components; time for data acquisition; data analysis parameters; platform requirements; etc). Are there redundant instrument/systems?
Are there any unusual sample requirements (blood or tissue, shipping, pre‐analytic processing, storage conditions, stability, etc)?
Describe the statistical analysis used; verification/validation of the routine.
What analytical verification/validation studies; clinical validation; correlation studies have been done? What method is used for comparison?
Are there other studies/publications using the method?
What is the intellectual property status? Are there other stakeholders in the technology?
What facilities are required to run the test? Will samples be run in an academic or CLIA‐certified laboratory; distributed or in a single location?

CLIA, Clinical Laboratory Improvement Amendment.

## MULTIPARAMETER CHARACTERIZATION OF CIRCULATING VS. SOLID TUMOR CELLS

Circulating tumor cells (CTCs) are viable tumor‐derived cells that exist in the peripheral blood of patients with cancer in very low concentrations (as low as ∼10^–8^/mL). The CTCs extravasate into the bloodstream and circulate; they may form secondary metastases, self‐seed, or remain in the circulatory system until clearance.^10^, ^11^, ^12^, ^13^ These cells are an accessible source of nonhematological tumor cells and, along with circulating nucleic acids, are components of what are termed liquid biopsies, which are increasingly being recognized as potentially valuable noninvasive tools for temporal characterization of a patient's tumor (including the constituent heterogeneity), because they can be isolated and characterized from a noninvasive blood draw.^14^, ^15^ One significant barrier to translation of CTC detection platforms to clinical use is the understanding of the relationship of biological signatures found in CTCs to those of solid tissue‐derived tumor cells to support the use of liquid biopsies in place of the more invasive and expensive solid tissue biopsies. One approach to overcome this hurdle is the analysis and comparison of biomarkers measured from isolated single cells from solid tissue and CTCs using a common platform. The HCDI working group worked with the Kuhn group at the University of Southern California and FNIH to design and fund a project, the High Definition Single Cell Analysis of Blood and Tissue Biopsies in Patients with Colorectal Cancer undergoing Hepatic Metastasectomy (HD‐SCA), to investigate the correlation between single‐cell‐based biological signatures of liquid biopsies, particularly CTCs and solid tumor tissue. One of the outcomes of this project will be to demonstrate the advantage of single cell, CTC‐based biomarkers to assist in laying the foundation for subsequent studies to define clinical utility of liquid biopsy‐based biomarkers.

**Figure 1:**
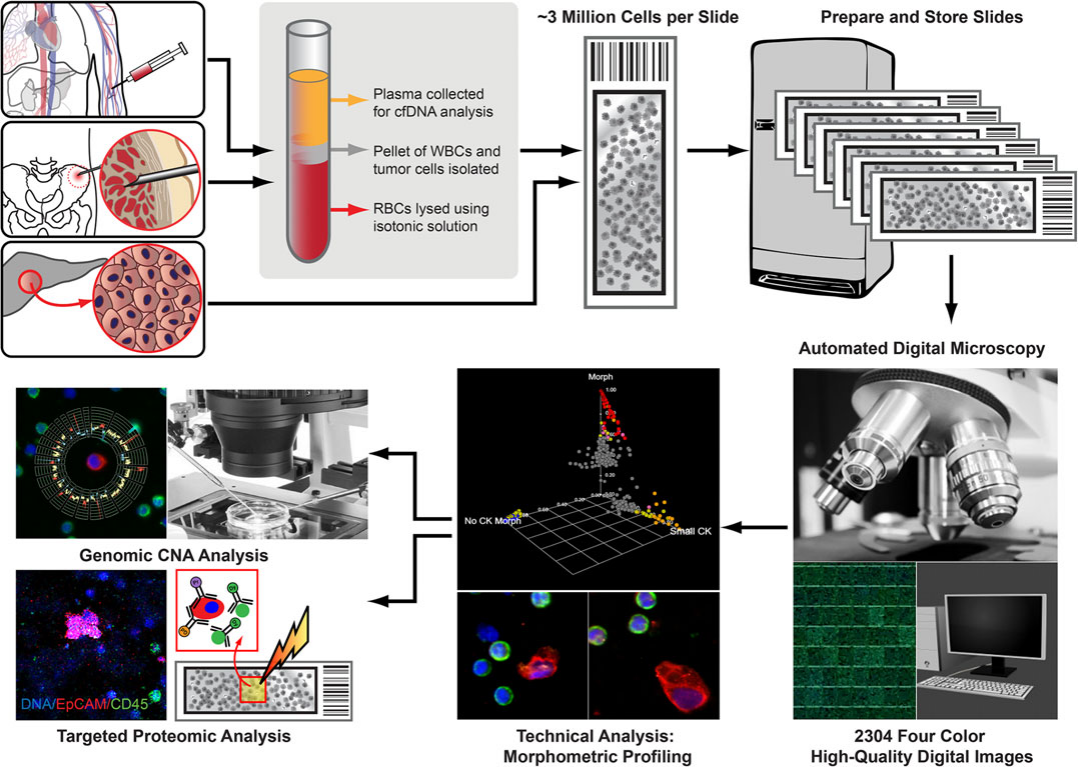
The high definition single cell analysis (HD‐SCA) generic temporal and spatial analysis. The HD‐SCA workflow is a single‐cell analysis system generating morphometric, proteomic, and genomic characterization of any rare cell from either liquid (blood draw or bone marrow aspirate) or solid tissue biopsy.^17^, ^19^, ^52^ Representative circulating tumor cells (CTCs) and solid tissue samples from patients with cancer are isolated and imaged using the same HD‐SCA system. Blood cells after red blood cell depletion and tissue cells obtained from touch preparations of either metastases or primary tumor are plated. Slides with nucleated blood cells and cell monolayers from touch preparations are immunofluorescently labeled in the same batch in three wavelengths, and the resultant stained slides are imaged at 40× magnification to generate high‐resolution digital images with detailed nuclear and cytoplasmic features for morphological cellular characteristics and protein expression. Captured CTCs are classified as CK+ (red), CD45– (green) cells of epithelial origin with an intact, nonapoptotic‐appearing nucleus by DAPI (blue) imaging, morphologically distinct from surrounding white blood cells by shape and/or size. Cells of interest can be picked individually and isolated for single‐cell genomic copy number alteration (CNA) or targeted proteomic analysis via imaging mass cytometry.

The HD‐SCA platform developed by Kuhn and colleagues^16^, ^17^ is a single‐cell direct imaging analysis system that can be used for morphometric, proteomic, and downstream genomic characterization of any rare cell from either the fluid phase or solid tissues with the flexibility to probe for any combination of markers needed to characterize specific populations of cells. This workflow then can be used to generate highly multiplexed data points derived from any combination of the morphologic, genomic, and proteomic (4–37 proteins with copy number variant and single nucleotide variation) measurements. As shown in **Figure** 1, all nucleated cells from a liquid biopsy are immunofluorescently labeled for high resolution digital imaging, providing detailed nuclear and cytoplasmic features for morphological and protein expression measurements. The location of a specific cell of interest on a slide is registered so that the cell can be selected for molecular analysis, including genomic sequencing, targeted proteomics, and other measurements.^17^, ^18^, ^19^ Moreover, a number of slides are generated from each patient sample so that each sample can be stained multiple times for analysis of different parameters.

## HYPERPLEXED FLUORESCENCE MEASUREMENTS OF THE CELLULAR AND SUBCELLULAR EXPRESSION LEVELS AND SPATIAL RELATIONSHIPS BETWEEN TUMOR BIOMARKERS IN TISSUE SECTIONS

A goal of high‐content and all image cytometry methods is to increase the number of biomarkers that can be measured within the same cells and tissues so that the complexity of intracellular interactions and intratumoral heterogeneities can be defined in greater molecular detail within the physical context or architecture of the tumor “system.” A number of approaches have been developed to address this goal from multiplexed fluorescence, with up to seven distinct fluorescent probes applied to the same sample, to hyperplexed fluorescence, using more than seven separate images of biomarkers in the same sample (reviewed in ref. 9). Extensive literature exists on the development of multiplexed and more recently hyperplexed fluorescence methods for tissue analyses, including instrumentation, software, and bioinformatics tools that can be used in the development of diagnostic tests.^9^, ^20^, ^21^, ^22^, ^23^ The ability to measure many parameters in large numbers of tissue samples has propelled these assays into the realm of big data challenges for the future.

**Figure 2:**
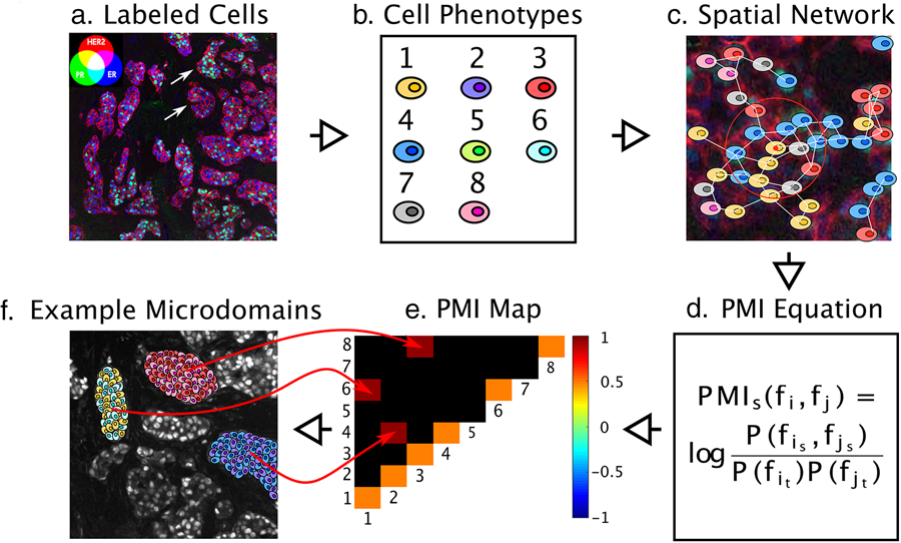
Pointwise mutual information (PMI) for quantifying spatial heterogeneity. (a) A pseudo‐colored multichannel fluorescence image labeled iteratively by the MultiOmyx platform is shown for an estrogen receptor (ER)+ invasive ductal carcinoma from a tissue microarray. Three biomarker channels were used to demonstrate the approach: HER2 (red), ER (blue), and PR (green), although this method can be scaled for >50 biomarkers. Areas of PR/ER co‐activation will appear in cyan, HER2/ER co‐activation in magenta, and PR/HER2 co‐activation in yellow. The upper and lower arrows indicate heterogeneous tumor microdomains with higher than average ER+/PR+ phenotyped cells and mostly ER+ cells, respectively. (**b**) Machine learning methods can be used to identify dominant cellular phenotypes from biomarker expression patterns over an entire tissue microarray, which in this case were eight. Each cell is then classified with the most similar dominant phenotype. (**c**) In order to represent the tumor topology, a spatial network of the cells in each tissue microarray spot or whole tissue section is constructed, in which each cell has the ability to communicate with nearby cells up to a certain limit, 250 μm,^59^ and the communication propensity is assumed to be inversely proportional to the cellular distance. (**d**) PMI quantifies the statistical associations, both linear and non‐linear, between each pair of cellular phenotypes. In particular, PMI calculates the logarithmic joint probability of finding a particular pair of cellular phenotypes occurring in close proximity, relative to the probability of these phenotypes co‐occurring at random. (**e**) By referencing a specific interaction pair in the PMI plot, one can interrogate the network subgraphs/motifs that contribute to the PMI dependencies. A PMI map with strong diagonal entries and weak off‐diagonal entries describes a globally heterogeneous but locally homogeneous tumor. On the contrary, a PMI map with strong off‐diagonal entries describes a tumor that is locally heterogeneous. (**f**) An example TMA spot with three locally heterogeneous tumor microdomains denoted by the off‐diagonal entries in the PMI map, containing phenotypes 1 and 6, 2 and 4, and 3 and 8. PMI maps can also portray anti‐associations (e.g., if phenotype 1 never occurs spatially near phenotype 3). The ensemble of associations and anti‐associations of varying intensities along or off the diagonal represent the true complexity of tumor images in a format that can be summarized and interrogated. PMI maps are predicted to become diagnostic and prognostic biomarkers.

One platform that exemplifies this approach evolved in collaboration among General Electric Global Research and the Taylor/Chennubhotla Laboratories at The Drug Discovery Institute, and the Department of Computational and Systems Biology at the University of Pittsburgh (**Figure** 2). MxIF is a fluorescence imaging technology that allows up to 60 fluorescent protein biomarkers, typically antibodies, to be analyzed *in situ* on a single formalin‐fixed, paraffin‐embedded tissue section.^24^ Using single or multiplexed (from 2–7 biomarkers) or hyperplexed (>7 biomarkers), the expression levels and subcellular locations of proteins can be quantified in tissue sections. DNA and RNA fluorescence *in situ* hybridization (FISH) may also be conducted on the same sample, thus providing additional ‐omic measurements. MxIF involves a series of sequential labeling, imaging, and dye inactivation steps so that a large number of different biomarkers can be measured with only a few fluorophores. Spatial statistical methods are then applied to the digital images to define the extent of heterogeneity and to identify tumor microdomains.^25^ The single cell analysis capability was originally demonstrated in the analysis of 700 patients with colorectal cancer with differing phosphorylation of ribosomal protein S6.^24^ Mutually exclusive phosphorylation patterns of the two canonical substrates of mTORC1 (S6K1 and 4E‐BP1) were observed in individual cells, large regions of most tumors, and in distinct cell lineages, demonstrating differential pathway activation. In the research setting, MxIF has also been applied to evaluate signaling pathway changes in mouse salivary glands,^26^ estrogen receptor (ER), progesterone receptor (PR), human epidermal growth factor receptor 2 (HER2), and Ki67 colocalization in breast cancer,^27^ and other single‐cell analyses.^28^, ^29^

The future of targeted multiplexed and hyperplexed fluorescence using platforms, such as MxIF, is very promising for basic research, including defining pathways of cancer evolution using “spatial proteomics” to directly map pathways inferred from genomics and proteomics approaches, and defining the microdomains of cancer within the native architecture, stromal and immune cell interactions that influence drug treatments, drug discovery/development, and diagnostics (**Figure** 2). The platform is valuable for identifying the optimal/minimal combination of biomarkers that could be incorporated into a smaller number of biomarkers for multiplexed tissue‐based diagnostic tests and biomarkers for drug development.^30^ Hyperplexed fluorescence with the MxIF using panels of biomarkers tailored to the cell types will be an important platform to define the functional interplay between the cells in the tumor microenvironment and to define the response to therapeutics when combined with automated machine learning software tools to identify and to quantify spatial patterns of biomarkers that reflect the heterogeneity of disease.

An alternative approach is imaging mass cytometry, as implemented on Fluidigm's Hyperion Imaging System. Antibodies are mass‐tagged with metal labels and, after incubation of liquid or solid biopsy samples on standard microscope slides, a region of interest is selected, laser ablated, and the atomized sample injected into a time of flight mass spectrometer. By calculating the slide position, this approach yields a 1 micron spatial resolution of the sample with 4–37 or more proteins simultaneously. This concept is currently being integrated into various basic science and clinical research workflows.

## UNIQUE CHALLENGES AND OPPORTUNITIES POSED BY SINGLE‐CELL ANALYSIS VARIABILITY

Single‐cell science attempts to measure the biological heterogeneity across cell populations to provide a quantitative description of the overall biological state or disease state. Biological heterogeneity can be manifested in a population of cells by the observation of a range of phenotypes even when the population of cells and the environment are nominally homogeneous. This heterogeneity can be due to stochastic fluctuations in molecular events, as well as extrinsic effects, including the effect of nearby cells.^31^ Biological heterogeneity, and the dynamics of how heterogeneity arises in a population, can be used to develop theoretical constructs for understanding control mechanisms and predicting population dynamics,^32^, ^33^ and provide a better understanding of intracellular pathways, control systems, and mechanisms that determine disease progression.

Biological heterogeneity cannot be assessed unless the technical variability in the measurement can be independently determined. Analytical methods, such as Western blots or enzyme‐linked immunosorbent assays or other solution‐based assays, provide a single value that represents an average over all the cells in the population. When using such techniques, single‐cell data that report on biological heterogeneity are lost, although replicates of the experiment can provide information about measurement variability. Single‐cell methods for transcriptomics can provide information about biological variability between cells, but the data are convoluted with measurement variability unless duplicate or triplicate measurements can be made on each cell. This is often impossible for single cell transcriptomics; replicate measurements are not typically accessible because of the quantity of samples and the limits on the sensitivity of the technique. Determining how to best address measurement uncertainties under this situation becomes a challenge. A number of approaches are being considered for assuring confidence in these measurements, including the use of the External RNA Controls Consortium (ERCC) spike‐in material^34^, ^35^ to provide a metric of accuracy for those known sequences. Other approaches include the use of Bayesian statistics to assess real differences in the presence of dropout events^36^ that occur due to the low amount of RNA in cell samples and result in detection of the gene in some cells and not in others. When the apparent heterogeneity in gene expression is simply due to technical issues, the data can lead to erroneous conclusions of biological heterogeneity. Other methods include the use of unique molecular identifiers in the primers for counting individual transcripts.^37^ In cases in which replicate measurements can be performed, such as has been shown with single cell proteomics,^38^ duplicate proteomic measurements from individual cells can be performed. Such replicate measurements can result in quantification of the technical error in the measurement, and unambiguous determination of whether the values measured from one cell are significantly different from the values measured for another cell.

Technologies, such as nondestructive microscopic imaging of individual cells, can have an advantage over other analytical methods, because it is possible to generate multiple samplings of the same field of view to provide a measure of instrument noise, such as shot noise, lamp fluctuations, and photobleaching. An automated routine that operates within open source software provides an easy way to qualify instrument performance to assess such operational figures of merit and to provide instrument calibration.^39^ The relative contribution of these sources of uncertainty can be compared with other aspects of the measurement and determined to be significant contributors to be quantified, or not significant contributors and can be safely ignored. Other aspects of the measurement that can add significantly to uncertainty include sampling, handling, and reagents. The aggregate of these contributions can be determined by comparison of the distribution of responses provided by replicate measurements as described in ref. 40, in which the width of the distribution of cell phenotype reflects biological heterogeneity and the variation between replicate measurements of the population distribution reflects measurement uncertainty. Using sufficient care to quantify measurement uncertainty allows deconvolution of biological heterogeneity and measurement uncertainty, and biological heterogeneity can be unambiguously determined. Accurate quantification of heterogeneity requires sampling of the appropriate number of cells; the more disperse the population phenotype the larger the number of cells that must be sampled to accurately represent the population.

Time‐resolved dynamic data on individual cells within a living cell population are uniquely accessible through imaging. Examining differences in dynamics of processes, such as promoter activation in a large number of individual cells, provides determination of variations in rates of fluctuations in cellular responses and epigenetic effects, and assists in choosing appropriate theoretical treatments.^33^ Dynamic data can also confirm stability in gene expression, such as in stem cell colonies, and the spatial location of the expressed gene within colonies. Such analysis can also allow detection of rare events.^41^ Longer‐term time‐dependent measurements of cell populations by imaging or flow cytometry can indicate whether population heterogeneity is the result of stochastic fluctuations within individual cells or is due to genomic differences within the population. A distribution in cellular responses within a population due to stochastic fluctuations will be stable or stationary over time. In this case, a selected “subpopulation” will relax back to the original broad distribution after a number of passages because each cell belongs to the same distribution of probable phenotypes.^33^ A population that is composed of true subpopulations that are genetically distinct will diverge according to the relative rates of proliferation of the genetically distinct cell populations.

Although measurement uncertainty cannot be eliminated, it can be mitigated by good experimental design and protocols, as discussed in **Table** 3. Mitigation of measurement uncertainty can be achieved in single‐cell measurements by appropriate control experiments in the evaluation of the single‐cell barcode chip for proteomics analysis.^42^ This group utilized well‐characterized recombinant proteins for calibration and defining the dynamic range of different protein assays and simulation based on known physical principles applied to the device to estimate measurement errors from locations of cells.

**Table 3:** Single cell measurement challenges and strategies for reducing uncertainty and increasing confidence

Challenge		Strategy
Measurements of biological response to environmental conditions		Measure sufficient numbers of cells to assure adequate sampling of population diversity (heterogeneity)
		Use appropriate statistics for comparison (e.g., cumulative distributions, not means)
		Both the mean response and the shape of the distribution of responses may change in response to treatment.
		Use appropriate positive and negative controls.
		Compare the results from orthogonal analytical methods: different methods should return similar responses.
		Measure response function (concentration or time dependence) to test for a systematic effect.
Distinguish inherent biological heterogeneity from measurement variability	Measurement variability	Quantify the uncertainty due to variability (e.g., SD) in the measured value due to instrument response. Measure within day (repeatability) and day‐to‐day (reproducibility).
		Test the sources of measurement variability (technicians, reagents, environment, algorithms, protocols), and try to mitigate them.
		Quantify the variation in results from the same sample on different platforms
	Biological heterogeneity due to stochastic fluctuations	Test the stability of the distribution of the population characteristic or phenotype.
		Measure similar distributions from repeated measurements of the population over long time intervals
		Sorted “subpopulations” will relax over time in culture to a stable distribution similar to the original distribution
		“Subpopulations” are genetically identical
	Biological heterogeneity due to genetic/genomic differences	Population phenotypic heterogeneity diverges over time in culture
		Subpopulations have transcriptomic and genomic differences
Minimize uncertainty in measurement variability		Assess instrument performance with benchmarking materials for signal to noise, linearity of response, limit of detection and saturation
		Use control materials (e.g., spike‐in RNA into transcriptomic samples) to test and compare assay platform response and to assess technical proficiency
		Use control materials to test and optimize protocols for accuracy, precision, sufficient dynamic range, sensitivity, specificity, and robustness to small protocol changes
		Test and compare algorithms for robustness and accuracy against ground truth (if available)

## VALIDATION AND REGULATORY CONSIDERATIONS

Although tests that leverage CTC platforms (i.e., platforms or systems that identify and isolate CTCs; the entire *in vitro* diagnostic (IVD) will be referred to as a “CTC test”) remain largely in the research and development phase, their diagnostic potential is clearly on the horizon. Widespread adoption of these tests in the clinic will require studies to demonstrate analytical and clinical validity for appropriate marketing authorization,^43^, ^44^ as well as clinical utility studies for adoption and reimbursement.^45^ Early consideration of regulatory requirements may make a large difference in reducing later potential regulatory challenges, many of which may be related to the platforms used to isolate CTCs or, if applicable, the high content methodology used for CTC analysis.

When reviewing a premarket submission for an IVD, the US Food and Drug Administration (FDA) typically evaluates data establishing the analytical and clinical validity of the IVD in the context of its proposed intended use. The types of studies necessary for marketing authorization will depend on the details of the diagnostic platform that is eventually developed. An open question is: what role will high‐content systems play in future diagnostic tests?

Currently, research using high‐content assays that use CTC platforms aim to identify new biomarkers for CTCs. If a discrete number of biomarkers are identified, IVDs that use more traditional techniques (e.g., antibody staining, polymerase chain reaction, etc) to detect those same biomarkers may be developed for a specific clinical use. In this scenario, the analytical and clinical validity of the assay could be demonstrated for each analyte that is detected. To date, the only CTC test that has been authorized by the FDA is the CellSearch Circulating Tumor Cell Kit, which is intended for the enumeration of CD45‐ EpCAM+ CK+ CTCs in whole blood for monitoring patients with metastatic breast, prostate, and colorectal cancer (see ref. 46 and related IVDs). The review of this product involved evaluation of the analytical and clinical validity of a well‐defined analyte (e.g., a specific biomolecule or set of biomolecules) for specific clinical conditions.

Another possibility may be the development of a clinical test that produces as its reportable output high content data from single cells with too many targets to validate independently. For instance, genome sequencing of single CTCs may help address issues of tumor heterogeneity in a patient with cancer when selecting an appropriate therapy (e.g., identifying rare cells that carry actionable somatic mutations). In such cases, special challenges related to the unique features of high‐content tests will need to be addressed, including the demonstration of analytical and clinical validation of high‐density data from rapidly evolving technologies; the possibility of unlimited (unique) results, with unknown clinical significance that are, therefore, open to interpretation; and the identification and availability of appropriate reference methods and clinical samples for demonstration of analytical reliability and variation across all possible outputs.

In either case, it will be important for developers to plan ahead on how to demonstrate analytical and clinical performance for regulatory clearance or approval. Below, we outline some of the key issues that should be considered as early as possible.

### Analytical validity

Analytical validity is the ability of a test to measure or detect a particular analyte (e.g., analytical specificity, limit of detection, analytical accuracy, precision, and robustness). Traditionally, for any type of assay that simultaneously measures multiple analytes, manufacturers must demonstrate that the test is analytically valid for each analyte. Because high‐content tests are designed to simultaneously assess a very large number of analytes, a complete demonstration of analytical validity may not be feasible. In some such cases, the FDA has allowed representative analytical performance encompassing the type of variants and analytical challenges that could be encountered during use of the instrument or assay, and inferred overall performance from this representative subset (e.g., Illumina MiSeqDx Cystic Fibrosis Clinical Sequencing Assay).^47^

### Clinical validity

Clinical validity is the association between the test result and a disease or condition, and is commonly described in terms of diagnostic accuracy. Test performance is commonly reported as clinical sensitivity and specificity, and/or positive or negative predictive value. Clinical validity for IVDs can be established using a variety of approaches, such as clinical studies or from evidence in the literature. This is often not possible for high‐content assays. Because they can simultaneously detect a large number of markers, such assays frequently detect rare or novel biological variation. Therefore, high‐content data can often be used to divide patients with a given diagnosis into small, or even private, subsets defined by specific markers (e.g., rare genetic variants). With very low prevalence markers, it is difficult to perform clinical studies to definitively demonstrate clinical validity, and published evidence may be limited or not available. In such cases, other sources of evidence may be considered, provided the evidence is appropriate for the intended use of the IVD. For instance, one strategy that was successful in the clearance of the Illumina MiSeqDX Cystic Fibrosis 139‐Variant Assay^48^ was to leverage information from the cystic fibrosis transmembrane conductance regulator CFTR2 database^49^ which is a high quality, well curated database that aggregates evidence from patients around the globe to establish the link between specific variants in the *CFTR* gene and the disease. The applicability of this strategy to other IVDs will depend on a number of factors, and may not be suitable for every situation.

The following additional issues should be considered for regulatory clearance or approval of IVDs incorporating single‐cell platforms, whether or not they included a high‐content component.

### Pre‐analytical variables

The term “pre‐analytical” variables refers to all factors that may affect a specimen or sample before it enters the analytical process. No matter how complete analytical and clinical validation for an assay is, if there is not confidence that the sample being analyzed actually reflects what is happening in the patient, then the results will be meaningless. Often times, especially in oncology and related fields, specimen collection, handling, and processing (CHP) variables are the most important of these pre‐analytical variables to consider. For example, what conditions (e.g., ischemia time) will be present at the time of surgery/resection? What will be the temperature range used for sample processing, shipping, and storage? In certain cases, it may be beneficial to undergo certain biospecimen‐based or pre‐analytical variable experiments to define the optimal collection, handling, and processing variables for the system, and ultimately produce best‐practice guidelines that the rest of the field can use.

### Specimens

Early planning should also take into account the need for adequate specimens that represent the analytes to be detected by the test. Given that high‐content assays will likely identify rare markers, it is often not possible to obtain specimens representing all possible analytes that could be detected. In such cases, it may be possible to use contrived samples in lieu of some patient specimens, provided there are data to demonstrate that the operational behavior of the contrived samples is similar to that of clinical specimens. Where applicable, the need for separate training and validation sets should be factored in.

### Reference materials and performance standards

One of the common challenges when developing new high‐content tests is the lack of available reference materials and performance standards for assessing analytical and clinical error rates. For this reason, collaborative efforts involving multiple stakeholders, including the FDA, National Institutes of Health, the National Institute of Standards and Technology (NIST), the Centers for Disease Control and Prevention (CDC), academia, and industry are critical for development of performance standards and reference materials for all types of next‐generation platforms, including both imaging and nonimaging methods, and for standardizing file formats and other aspects of informatics associated with these platforms. Such efforts are already beginning to yield results. For instance, the high‐confidence calls produced by the National Institute of Standards and Technology through the Genome in a Bottle (GIAB) Consortium^50^ are widely used as standard reference materials for validation of next‐generation sequencing‐based tests. However, those sequences do not currently cover many of the medically relevant variants that will be detected using genomic tests and additional work is being done by the GIAB Consortium, the FNIH, and other efforts to address this. For other technologies, it is important for developers to assess whether reference materials and standards are available and, if not, what alternative strategies can be used to establish analytical validity in their absence. In the absence of reference materials, confirmation of results by a validated orthogonal method may be used to benchmark test performance.

## DISCUSSION

It is becoming apparent that cancer is an evolutionary process of genetic, epigenetic, molecular, and physical‐based alterations that manifests as heterogeneity across a tissue or development of resistance to therapeutic interventions over time. Clinical treatment decisions are often made based upon bulk measures of bone marrow blasts or size of a tumor mass on a computed tomography scan, leaving a vast amount of biologic information on the table that could theoretically be leveraged for the good of the patient. The ability to sample the cancer cells throughout the course of treatment and disease progression, and assay multiple functional changes simultaneously with the platforms, such as those described in this paper, may enable better and quicker detection of these changes and the ability to improve therapeutic interventions for disease management. The platforms profiled here are examples of instruments that translate high‐content information out of the research laboratory to the clinical research setting for characterization and monitoring of single cells in tissue or blood. They go beyond enumeration of assay end points to enable profiling of heterogeneous cell populations. As such, the systems could be exploited in the drug development and clinical arenas not only for *in vitro* evaluations guiding clinical decisions, but also *ex vivo* studies, such as assessing target activity, probing for clinically relevant targets, and characterizing cancer cell and tumor heterogeneity.

Because these multiparameter systems can involve complicated analytic signatures, one of the keys to translation of these new tools into the clinic will be in the careful attention to deconvolution of biological and instrument variability, and demonstration of analytical performance. In addition, demonstration of the biological significance and usefulness of an analysis is key. Furthermore, it is important to pay thoughtful attention to a developmental regulatory strategy, including early determination of the intended use of the platform, which will lead to successful development, validation, regulatory authorization, and clinical use. The process of making such multi‐analyte platforms and complex analysis more generally available, such as through commercial clinical assay systems, may present interesting challenges. An understanding of the path to market for next‐generation sequencing platforms and other complex clinical systems^51^ can inform that process, as more of these multiparameter, complex technological platforms will undoubtedly make their way to into the clinic.

## Conflict of Interest

D.L.T. is a co‐founder of Spintellex, a computational pathology company. P.K. is a shareholder, royalty recipient, and advisor of Epic Sciences, which has licensed the HD‐SCA technology from The Scripps Research Institute.
